# Inhibition of the Formation In Vitro of Putatively Carcinogenic Metabolites Derived from *S. haematobium* and *O. viverrini* by Combination of Drugs with Antioxidants

**DOI:** 10.3390/molecules24213842

**Published:** 2019-10-25

**Authors:** Maria João Gouveia, Verónica Nogueira, Bruno Araújo, Fátima Gärtner, Nuno Vale

**Affiliations:** 1 Laboratory of Pharmacology, Department of Drug Sciences, Faculty of Pharmacy, University of Porto, Rua de Jorge Viterbo Ferreira 228, 4050-313 Porto, Portugal; mariajoaogouveia@gmail.com (M.J.G.); veronicaanogueira54@hotmail.com (V.N.); brunoaraujo61@hormail.com (B.A.); 2 Department of Molecular Pathology and Immnunology, Institute of Biomedical Sciences Abel Salazar (ICBAS), University of Porto, Rua de Jorge Viterbo Ferreira 228, 4050-313 Porto, Portugal; fgartner@ipatimup.pt; 3 Center for the Study of Animal Science, CECA-ICETA, University of Porto, Praça Gomes Teixeira Apartado 55142, 4051-401 Porto, Portugal; 4 Institute of Molecular Pathology and Immunology of the University of Porto (IPATIMUP), Rua Júlio Amaral de Carvalho, 45, 4200-135 Porto, Portugal; 5 i3S, Instituto de Investigação e Inovação em Saúde, university of Porto, Rua Alfredo Allen 208, 4200-135 Porto, Portugal

**Keywords:** helminth infections, carcinogenesis, DNA adducts, CYP enzymes, antioxidants, drug combination, drug repurposing

## Abstract

Infections caused by *Schistosoma haematobium* and *Opisthorchis viverrini* are classified as carcinogenic. Although carcinogenesis might be a multifactorial process, it has been postulated that these helminth produce/excrete oxysterols and estrogen-like metabolites that might act as initiators of their infection-associated carcinogenesis. Current treatment and control of these infections rely on a single drug, praziquantel, that mainly targets the parasites and not the pathologies related to the infection including cancer. Thus, there is a need to search for novel therapeutic alternatives that might include combinations of drugs and drug repurposing. Based on these concepts, we propose a novel therapeutic strategy that combines drugs with molecule antioxidants. We evaluate the efficacy of a novel therapeutic strategy to prevent the formation of putative carcinogenic metabolites precursors and DNA adducts. Firstly, we used a methodology previously established to synthesize metabolites precursors and DNA adducts in the presence of CYP450. Then, we evaluated the inhibition of their formation induced by drugs and antioxidants alone or in combination. Drugs and resveratrol alone did not show a significant inhibitory effect while *N*-acetylcysteine inhibited the formation of most metabolite precursors and DNA adducts. Moreover, the combinations of classical drugs with antioxidants were more effective rather than compounds alone. This strategy might be a valuable tool to prevent the initiation of helminth infection-associated carcinogenesis.

## 1. Introduction

Helminths are the most common infectious agents of humans in developing countries affecting about one-third of the world’s population [1]. About 20% of cancers in these regions are caused by infections [1,2], including schistosomiasis, one of the major neglected tropical diseases and opisthorchiasis [3]. Their causative agents, *Schistosoma haematobium* and *Opisthorchis viverrini* are considered as biological carcinogenic agents of group 1, i.e., its infection leads to squamous cell carcinoma (SCC) of the urinary bladder and cholangiocarcinoma (CCA), bile duct cancer [4]. It is estimated that 5000 deaths annually occurred in Northeast Thailand derived from *O. viverrini*-associated cholangiocarcinoma [5,6]. By 2006, the incidence of schistosomiasis haematobia-associated bladder cancer is 3–4 cancer per 100,000 annually but it is plausible to believe that this number is underrated [7]. Notably, case reports from highly endemic regions indicate that patients with schistosomiasis may develop bladder cancer in their first or second decade of life [8]. However, the molecular and cellular mechanism linking these infections with associated cancers remain elusive. Recently, our group indicate that schistosomes and opisthorchiids produce/excrete estrogen- and oxysterol-like metabolites that might react with host DNA leading to its oxidation or forming depurinating DNA adducts. This interaction induces mutations in the genome of adjacent host tissues that could trigger the carcinogenesis. Therefore, the metabolites derived from helminths might be considered as initiators of carcinogenesis [2,9]. Oxysterols and estrogen-like metabolites were identified and characterized through high-performance liquid chromatography coupled with mass spectrometry (LC-MS/MS) in sera, urine from individuals with urogenital schistosomiasis (UGS) and bladder cancer [10,11,12], and in developmental stages of *S. haematobium* and *O. viverrini* [13]. More recently, similar metabolites and evidence of their interaction with host DNA were identified in biofluids from hamsters experimentally infected with *O. felineus* reinforcing the notion that parasite might also have a potential carcinogenic similar to *O. viverrini* [14]. A pertinent question arises: how are these metabolites derived from helminths formed? Previously our research group performed in vitro assays to observe the interaction of metabolites, similar to those derived from helminths and associated with schistosomiasis and opisthorchiasis-associated cancers, with DNA in presence or absence of CYP450 isoforms [15]. The CYP450 isoforms as CYP1A1, 2E1 and 3A4 are related to the metabolism of chemical carcinogens associated with several human cancers [16]. Also, they are responsible for the metabolism of most drugs [17,18]. Genomic studies identified members of the CYP450 family genes in schistosome and opisthorchiids [19,20,21].

**Scheme 1:**
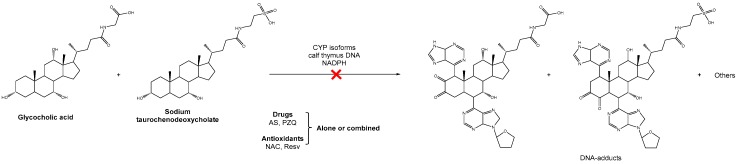
Schematic representation of the inhibition by drugs and antioxidants, alone or in combination, of the formation of putatively carcinogenic metabolites derived from schistosomes and opisthorchiids and its associated DNA adducts.

The current treatment against these helminth diseases relies on a single drug, praziquantel (PZQ) that mainly targets the parasite [22]. PZQ is effective against all forms of schistosome and opisthorchiids and has a safety profile. Nevertheless, the drug has major drawbacks including inefficiency against juvenile forms of parasites, and alone they do not prevent/ameliorate pathologies associate with infection [23]. In addition, there is a legitimate concern of resistance to PZQ due to its extensive use [24,25]. Given that parasites produce putative carcinogenic metabolites, the novel therapeutic strategy should not only target the parasite but also prevent their formation of these helminths derived metabolites and ultimately block carcinogenesis. With this in mind, we proposed a novel therapeutic strategy-based on drug repurposing and rational combination of drugs with antioxidants. These strategies have several advantages such as reducing costs and time of drug development, achieving an additive/synergistic therapeutic effect allowing lower doses and thereby reducing adverse effects and minimizing or delaying the onset of drug resistance [26,27]. Antioxidants presented interesting biological properties which might prevent DNA damage, block carcinogenesis, improve and ameliorate histopathological parameters, and are pharmacological safe agents [28,29,30,31,32], rendering them as interesting candidates to use in therapy against these diseases. Here, we conducted in vitro assays to evaluate if the novel therapeutic strategy counteracts the formation of these helminth derived metabolites. Thus, we used PZQ (anthelmintic), artesunate (AS) as drug repurposed, antioxidants *N*-acetylcysteine (NAC) and resveratrol (Resv) either alone or in combination (Scheme 1). Impressively, not only did antioxidants alone almost completely inhibit the formation of several precursors and DNA adduct in vitro, but when combined with drugs they potentiated their inhibitory activity.

## 2. Results

Previously, our research group demonstrated the ability of the compounds depicted in Scheme 1 to interact with DNA in vitro leading to the formation of DNA adducts. It should be noted that the compounds are similar to those detected in developmental stages of schistosomes and opisthorchiids [15]. Herein, we evaluated the potential inhibition of its formation using the new therapeutic strategy.

### 2.1. Formation of Metabolites Precursors and DNA Adducts in Presence of CYP450

Due to difficulty in obtained metabolites from extracts of parasites, we selected two commercial compounds similar to metabolites derived from helminths. Previously, we observed that the production of metabolites and DNA-adducts were independent of CYP450 enzymes [15]. In this study, we included the CYP450 isoforms and the reaction mixtures as they may be involved in drug and antioxidant metabolism and consequently affect their activity [17,18]. Considering our previous assay, a period of 72 h of the reaction was selected for this study.

**Figure 1:**
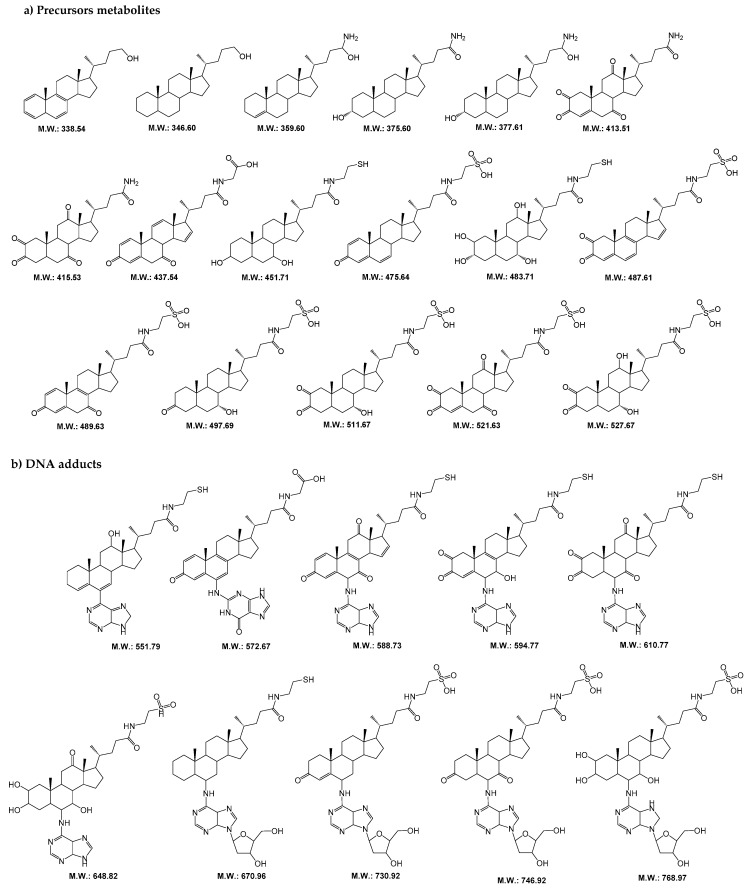
Postulated molecular structures for (**a**) metabolites precursors and (**b**) DNA adducts originated from the reaction of glycocholic acid and taurochenodeoxycholate sodium with calf thymus DNA in vitro in presence of CYP450 isoforms. (M.W., molecular weight).

Initially, we performed an assay to identify the metabolites precursors and DNA adducts formed in presence of starting compounds (glycocholic acid and taurochenodeoxycholate sodium) and in the presence or absence of CYP450 isoforms. Then, we compared the LC-MS/MS data obtained for these two samples (data not shown) and selected the exclusive metabolites precursors and their DNA adducts formed in the presence of CYP450 isoforms. From this selection, we obtained a total of 29 compounds in which 17 correspond to precursor metabolites (*m*/*z* 330–500) and 12 to DNA adducts (*m*/*z* 500–770). Their postulated molecular structural is depicted in Figure 1. It is important to note that several of the metabolites and DNA adducts detected in these samples or the oxidized form [M + 16] are already associated with helminth infections caused by *S. haematobium* and *O. viverrini* [10,11,12,13,14].

### 2.2. Novel Therapeutic Approach Inhibited the Formation of Metabolites Precursors and DNA Adducts.

Following that, we evaluate if the novel therapeutic approach inhibits the formation of metabolites precursors and/or its DNA adducts previously synthesized. For that purpose, reaction mixtures containing starting compounds, CYP450 isoforms, and drugs or antioxidants alone or combined were performed in vitro as described in Section 4. The chromatographic profiles and *m*/*z* obtained by LC-MS/MS are depicted in Supplementary Figure S1. The number of *m*/*z* detected in the combination of drugs with antioxidants was more reduced than drugs and antioxidants alone (Supplementary Figure S1). The data obtained for all samples were compared with the list of metabolites and DNA adducts detected on control to evaluate the inhibition of their formation (Table 1).

**Figure 2:**
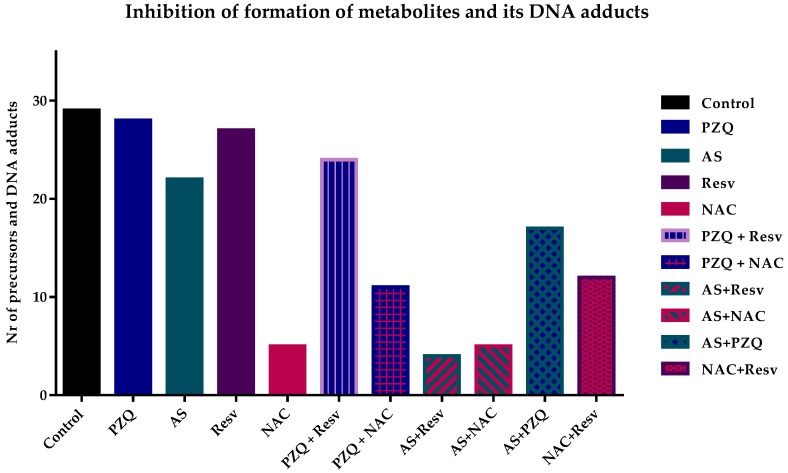
Graphical representation of the number of metabolite precursors and DNA adducts detected in different samples. In general, combination presented better results than compounds alone with AS + Resv inhibiting the formation most of metabolites precursors and DNA adducts. Additionally, antioxidant NAC also prevents the formation of them.

The drugs alone did not significantly inhibit the formation of precursor metabolites or DNA adducts (Table 1). Nevertheless, the inhibitory effect of AS was slightly higher compared to PZQ. AS inhibited the formation of three precursors and four DNA adducts while PZQ only inhibited the formation of three precursors and had no effect on the formation of DNA-adduct (Table 1). Regarding antioxidants alone, NAC was the most potent to inhibit the formation either of metabolites precursors or DNA adducts; it only detected three metabolites precursors (e.g., *m*/*z* 338.90, 346.87, 378.90) and three DNA adducts (e.g., *m*/*z* 572.79, 588.79, and 648.81). Resv presented a similar effect as PZQ inhibiting only five metabolites precursors and curiously did not affect the production of DNA adducts (Table 1 and Figure 2).

The inhibition of formation metabolites and DNA adducts was more pronounced when drug and antioxidants were combined (Table 1 and Figure 2). Combinations of AS with antioxidants were more effective in inhibiting their formation in comparison to PZQ plus antioxidants (Figure 2). Indeed, AS + Resv resulted in the highest inhibition presenting only three metabolites and DNA adduct detected on control (Table 1). This evidence suggests that the discrete effect of drug and antioxidants alone is potentiated when compounds are combined leading to a potential synergistic effect. In the case of AS + NAC, the inhibitory effect was similar to those observed for NAC alone (7 vs. 6) suggesting that the effect observed in combination derived from antioxidants (Figure 2). The inhibitory effect of PZQ plus antioxidants was slightly better than the drug alone. Of these combinations, PZQ + NAC had a better result in comparison to PZQ + Resv. The combination of PZQ + NAC inhibited the formation of 17 metabolites precursors/DNA adduct while PZQ + Resv only inhibited the formation of 7 compounds. The inhibitory effect observed in PZQ + NAC most likely derived from NAC, nevertheless, in combination the effect was not so pronounced as NAC alone. The combination PZQ + Resv, that inhibited the formation of 7 metabolites precursors or DNA adducts, was slightly better than both compounds alone; PZQ inhibited 7 and Resv 5 (Figure 2).

Additionally, we evaluated the inhibitory effect of the formation of the combinations of a drug plus drug (AS + PZQ), and antioxidant plus antioxidant (Resv + NAC). The combination of AS + PZQ achieved better results than drugs alone inhibiting the formation of a higher number of compounds (13), either precursor’s metabolites or DNA adducts, in comparison to the drugs alone (Figure 2). Concerning the combination of NAC + Resv, it was possible to observe a decrease of precursors and DNA adducts more pronounced than Resv alone. However, the inhibitory effect induced by this combination was lower when compared to NAC alone. Nevertheless, the combination NAC + Resv was more efficient than AS + PZQ possibly due to the effect induced by NAC since this antioxidant alone inhibited the formation of most precursors’ metabolites and/or DNA adducts (Figure 2).

## 3. Discussion

Infections caused by carcinogenic agents *O. viverrini* and *S. haematobium* affect millions of people worldwide and are important in terms of mortality and morbidity. Bladder and bile duct cancers are a dire and frequent consequence associated with these infections [33,34]. Probably, the infection-associated with carcinogenesis is a multistep and multifactorial process [35]. Carcinogenesis might undergo a sequence of events that include a pathogenic stimulus, biological or chemical, followed by a chronic inflammation that leads to fibrosis and alters the cellular environment arising a pre-cancerous niche [36]. Regard to opisthorchiasis and schistosomiasis, it was postulated that these pathogens provide biological and chemical stimuli through the production of estrogen and oxysterol-like metabolites that might interact with host DNA triggering a cascade of events that culminate to develop of cancer [2,9]. In our point of view, the therapeutic strategies against these diseases not only should target the parasite but ultimately counteract the formation of these metabolites. Thus, we developed a novel therapeutic strategy based on drug repurposing and the combination of drugs with antioxidants. Previously, this novel strategy demonstrated to be effective against the developmental stages of schistosomes [37,38]. Here, we evaluated the effect of this combination of drugs with antioxidants in inhibition of the formation of metabolites precursors and their DNA adducts.

Recently we explored the generation of some of the metabolites and related DNA-adducts previously identified in the context of opisthorchiasis and schistosomiasis and their infection-associated cancers. In that study, we confirmed the ability of glycocholic acid and taurochenodeoxycholate sodium to interact with DNA leading to the formation of DNA adducts [15]. Their formation might be independent of the parasite CYP450, nonetheless, in the presence of isoforms either metabolites or DNA adducts were also detected. Based on the same methodology, here we synthesized metabolites precursors and DNA adducts of glycocholic acid and taurochenodeoxycholate sodium in presence of isoforms of CYP450. Despite that their formation may be independent of CYP450, this family of enzymes might play a role in the metabolism of drugs and interfere with their activity [17,18]. Through LC-MS/MS analysis we detected several oxidized forms of starting compounds and also their DNA-adducts that were originated during the reaction. The metabolites precursors and their DNA adducts detected here were similar to those observed in our previous work [15].

Impressively, drugs and antioxidants alone, and especially when combined, lead to a reduction of formation of either metabolites precursors or DNA adducts. In fact, it seems that the metabolism of drugs by CYP isoforms altered the drug activity. Among the drugs evaluated, PZQ achieved a lower reduction in their formation which might be related to the fact that PZQ is extensively metabolized by CYP 3A4 resulting in numerous mono- and dehydroxylated derivates [39]. Probably, these PZQ metabolites are less reactive and thus do not prevent the formation of the metabolite precursors and/or DNA adducts. In comparison to PZQ, AS leads to a more pronounced reduction of their formation. The drug is rapidly metabolized to its active form dihydroartemisinin through cleavage of hemisuccinate ester-linked to artemisinin [40]. This suggests that dihydroartemisinin might prevent the formation of some metabolites and/or DNA adducts. However, this metabolite of AS is also metabolized by CYP3A4 to an inactive form [40]. Thus, it is reasonably hypothesized that the conversion of AS to its inactive form reduces the drug capacity to prevent the formation of metabolites and/or precursors of DNA. This might explain why AS is capable of reducing their formation but not significantly. Regarding antioxidants alone, NAC presented the most pronounced inhibitory effect leading to almost complete inhibition of the formation of metabolites precursors/DNA adducts. In other studies, it was already demonstrated that NAC and Resv were able to inhibit the formation of catechol estrogen quinone (CEQ)-DNA [41]. The inhibition of the reaction between precursors metabolites and DNA by the action of these antioxidants might be related to the fact that these antioxidants react with electrophilic compounds, e.g., CEQ, preventing their reaction with DNA, and consequently inhibited the formation of DNA adducts [29,30,41]. Also, NAC reduces semiquinones to their catechol forms and indirectly prevents the formation of DNA adducts [29]. Similar to NAC, Resv also has several important characteristics in the context of carcinogenesis prevention. In studies using cell lines, Resv was demonstrated to be a quinone reductase (NQO1) inducer, and also is responsible for reducing the electrophilic compounds CEQ in its catechol form, modulating the activity of CYP1A1 that is responsible for catalyzing the oxidation of estrogen to catechol forms [30]. Curiously, in this study, Resv alone did not affect the production of metabolites precursors and its DNA adducts.

Generally, the inhibitory effect was more pronounced when drugs were combined with antioxidants. The combination that achieved better results was AS + Resv which might be related to the fact that Resv could inhibit the activity of isoform CYP3A4 [42]. Resv might block the metabolism of AS or dihydroartemisinin maintain its reactivity, thus, the drug can interact with metabolites precursors and inhibited the formation of DNA adducts. Indeed, it was possible to detect the active metabolite of AS, dihydroartemisinin ([M + H] 284.33, Supplementary Figure S1), in the analysis of a sample from AS + Resv by LC-MS/MS. The combination of PZQ + Resv achieved better results than compounds alone which might be explained similarly to AS + Resv. Regarding the combination of PZQ or AS with NAC, the inhibitory effect might derive from NAC. Nevertheless, the inhibition of formation induced by the combination was lower than NAC alone, which might suggest that NAC might interact with the parental drug, with potentiation of activity but decreasing the antioxidant power.

Interestingly the combinations of drug + drug and antioxidant + antioxidant were not as effective as combinations of drugs with antioxidants or NAC alone. Nevertheless, for AS + PZQ the reduction of metabolites/DNA adducts was more pronounced than compounds alone. These two drugs (at the same time) can be metabolized by the same enzyme, and can occur in competition and, so that, lead to a lower degree of metabolism of PZQ and AS. Thus, there is more availability of drugs to interact with the starting compounds and inhibit the formation of precursor metabolites or DNA adducts. The combination NAC + Resv demonstrated a better inhibitory effect than AS + PZQ which might be related to NAC activity. Nevertheless, NAC + Resv had better activity than Resv alone but not NAC alone suggesting the environment in which biomolecules antioxidants are might influence their antioxidant activity.

To conclude, we demonstrated that antioxidants such as NAC reduced the formation in vitro of most metabolites precursors and their DNA adducts that might be putatively carcinogenic. Despite this, the drugs alone did not induce a considerable reduction, but when combined with antioxidants increased its inhibitory effect. Thus, the novel therapeutic strategy should be pursued since it might be valuable to prevent the *O. viverrini* and *S. haematobium* infections-associated with carcinogenesis by counteracting the formation of putative carcinogenic metabolites derived from helminths. Future studies using informative cell lines should be undertaken not only to assess the carcinogenic effect of these metabolites but also to evaluate the effect of the new therapeutic strategy on inhibiting their formation in the cellular environment and its effect in vitro and in vivo.

## 4. Materials and Methods 

### 4.1. Reagents and Material

Acetonitrile (ACN) and formic acid (HF), HPLC grade, were obtained from Merck (Darmstadt, Germany). Glycocholic acid hydrated (G2878-100MG), praziquantel (P4668-1G), calf thymus DNA (D1501-100MG), nicotinamide adenine dinucleotide phosphate (NADPH, N7506-25MG), dimethyl sulfoxide (DMSO, D-5879) and CYPExpress™ 1A1 (MTOXCE1A1-250MG), 2E1 (MTOXCE2E1-250MG) and 3A4 (MTOXCE3A4-250MG) were purchased from Sigma/Merck (Sintra, Portugal). Taurochenodeoxycholate sodium (20275) and artesunate (AS, 11817) was purchased from Cayman Chemical (Ann Arbor, MI, USA). Resveratrol (sc-200808) was purchased from Santa Cruz Biotechnology (Dallas, TX, USA). Absolute ethanol 90% was purchased from Panreac.

### 4.2. Formation of Precursor Metabolites and DNA Adducts through Their Interaction with DNA In Vitro

The formation of precursors metabolites and DNA in vitro were performed as previously described [15]. Briefly, stock solutions of compounds glycocholic acid and taurochenodeoxycholate sodium were prepared in 100% DMSO. Then, the compounds at final concentration of 100 μM were incubated with CYP isoforms, CYP1A1, CYP2E1, CYP3A4 (0.2 μM) in the presence of 1.4 μM of NADPH and calf thymus DNA (3 mM) in 67 mM Na-K phosphate buffer (pH 7.2) in a total volume of 200 μL in 96 wells flat bottom plate (Nunclon, Roskilde, Denmark) at 37 °C for 72 h. Aliquots were collected following 72 h after the onset of the reaction. Afterwards, the reaction was ended following the addition of two volumes of cold absolute EtOH to precipitate DNA, which was recovered by centrifugation. Thereafter, 20 μL of the supernatant was subjected to analysis by liquid chromatography coupled to mass spectrometry (LC-MS/MS).

### 4.3. Inhibition of Formation of Metabolites Precursores and DNA Adducts by Novel Therapeutic Approach

The stock solutions of PZQ, AS, NAC, and Resv were prepared in 100% DMSO. The compounds at a final concentration of 50 μM were incubated with glycocholic acid, taurochenodeoxycholate sodium, CYP isoforms, calf thymus DNA in 67 mM Na-K phosphate buffer (pH 7.2) in a total volume of 200 μL at 37 °C for 72 h, as described above. When combined, the drugs and antioxidants were incubated at a constant ratio 1:1 with the same concentration of 50 μM. The constitution of each mixture reaction is depicted in Table 2. Aliquots were collected 72 h. The reactions were stopped through addition by EtOH, centrifugated, and analyzed as described above.

### 4.4. Evaluation of Inhibition of Precursores and DNA Adducts Formation by Liquid Chromatography Coupled with Mass Spectrometry (LC-MS/MS) In Vitro

Detection and identification of metabolites and related DNA-adducts by LC-MS/MS were conducted using LTQ Orbitrap XL mass spectrometer (Thermo Fischer Scientific, Bremen, Germany), fitted with an ultraviolet (UV) photodiode assay (PDA) detector as described elsewhere [15]. Briefly, the analysis of aliquots was undertaken by a single injection of 20 μL with an ACE Equivalence 5 C_18_ (75 mm × 3 mm internal diameter) column. The mobile phase consisted of 1% HF in water (A)/CAN (B) mixtures. Elution undergoes at a flow rate of 0.5 mL/min. Eluates were monitored for 8 min, run with mobile phase gradient started with 80% A and 20% B. Then, B was increased linearly to 55% B and 45% A over 5 min and returned to the starting point 5 to 8 min and equilibrated for one minute. Data were collected in positive electrospray ionization (ESI). The capillary voltage of the ESI was 28 kV, and with a temperature of 310 °C. Flow rates of the sheath gas and auxiliary gas (N_2_) were set to 40 and 10 (arbitrary units as provided by software settings), respectively, and the gas temperature was 275 °C.

## Figures and Tables

**Table 1:** Evaluation of inhibition of the formation of precursors metabolites and DNA adducts detected in the control sample by drugs and antioxidants alone and combined. The structures of these *m*/*z* compounds are represented in Figure 1. The tick and cross indicate the presence (√) or absence (×) of the correspondent metabolite or DNA adduct in the samples analyzed in this study.

	Samples									
*m*/*z*	PZQ	AS	NAC	Resv	PZQ + Resv	PZQ + NAC	AS + Resv	AS + NAC	PZQ + AS	NAC + Resv
338.90	√	√	√	×	√	√	×	×	×	√
346.87	×	×	√	×	×	×	×	×	√	×
362.93	×	√	×	√	√	×	×	×	×	×
376.86	√	√	×	√	×	×	×	×	×	√
378.90	√	×	√	√	√	×	√	√	√	√
414.90	×	√	×	√	√	×	×	×	×	×
416.91	√	√	×	√	√	×	×	×	√	√
436.88	√	√	×	√	√	×	×	×	√	√
452.85	√	√	×	√	√	×	×	×	×	√
474.83	√	√	×	√	√	×	×	×	√	×
482.20	√	×	×	×	√	√	×	×	√	√
488.31	√	√	×	×	√	×	×	×	√	×
490.79	√	√	×	√	×	×	×	×	×	×
496.81	√	√	×	√	√	√	×	×	√	√
512.79	√	√	×	√	√	×	×	√	×	×
522.29	√	√	×	×	×	√	×	×	×	×
528.84	√	√	×	√	√	×	×	√	×	√
550.83	√	√	×	√	√	×	×	×	√	×
572.79	√	×	√	√	√	√	×	×	×	×
588.79	√	√	√	√	√	√	√	√	√	√
594.78	√	√	×	√	√	√	×	×	√	×
610.77	√	√	×	√	√	√	×	×	×	×
626.81	√	√	×	√	√	×	×	×	×	√
632.83	√	√	×	√	√	√	×	×	×	√
648.81	√	×	√	√	×	×	×	×	√	×
670.79	√	×	×	√	√	×	√	√	√	×
730.81	√	√	×	√	×	√	×	×	√	×
746.78	√	×	×	√	√	√	×	√	√	×
768.70	√	√	×	√	×	×	×	√	√	×

**Abbreviations: *m*/*z***—ratio mass(m)/charge(z); **PZQ**—praziquantel; **AS**—artesunate; **Resv**—resveratrol; **NAC**—N-acetylcysteine; **PZQ + Resv**—combination of praziquantel plus resveratrol; **PZQ + NAC**—combination of praziquantel plus *N*-acetylcysteine; **AS + Resv**—combination of artesunate plus resveratrol; **AS + NAC**—combination of artesunate plus N-acetylcysteine; **PZQ + AS**—combination praziquantel plus artesunate; **NAC + Resv**—combination of N-acetylcysteine plus resveratrol.

**Table 2:** Constituents of the reaction mixtures used for evaluation of inhibition of precursor metabolites and DNA adducts formation by novel therapeutic approach.

	Glycocholic Acid	Taurocheno-Deoxycholate Sodium	NADPH	CYP Isoforms	DNA	PZQ	AS	Resv	NAC
**Control**	√	√	√	√	√	×	×	×	×
**PZQ**	√	√	√	√	√	√	×	×	×
**AS**	√	√	√	√	√	×	√	×	×
**Resv**	√	√	√	√	√	×	×	√	×
**NAC**	√	√	√	√	√	×	×	×	√
**PZQ + Resv**	√	√	√	√	√	√	×	√	×
**AS + Resv**	√	√	√	√	√	×	√	√	×
**PZQ + NAC**	√	√	√	√	√	√	×	×	√
**AS + NAC**	√	√	√	√	√	×	√	×	√
**AS + PZQ**	√	√	√	√	√	√	√	×	×
**NAC + Resv**	√	√	√	√	√	×	×	√	√

## Supplementary Materials

The following are available online, Figure S1: Mass spectra and *m*/*z* obtained for different samples analyzed by LC-MS/MS.
