# Supplementary material for: Inhibition of the Formation In Vitro of Putatively Carcinogenic Metabolites Derived from S. haematobium and O. viverrini by Combination of Drugs with Antioxidants
Source: Molecules. 2019 Oct 25;24(21):3842. doi: 10.3390/molecules24213842 (PMC6864706; doi:10.3390/molecules24213842)
Supplement: Supplementary file 1 [file molecules-24-03842-s001.pdf]

Figure S1: Mass spectra and m/z obtained for different samples analyzed by LC-MS/MS.

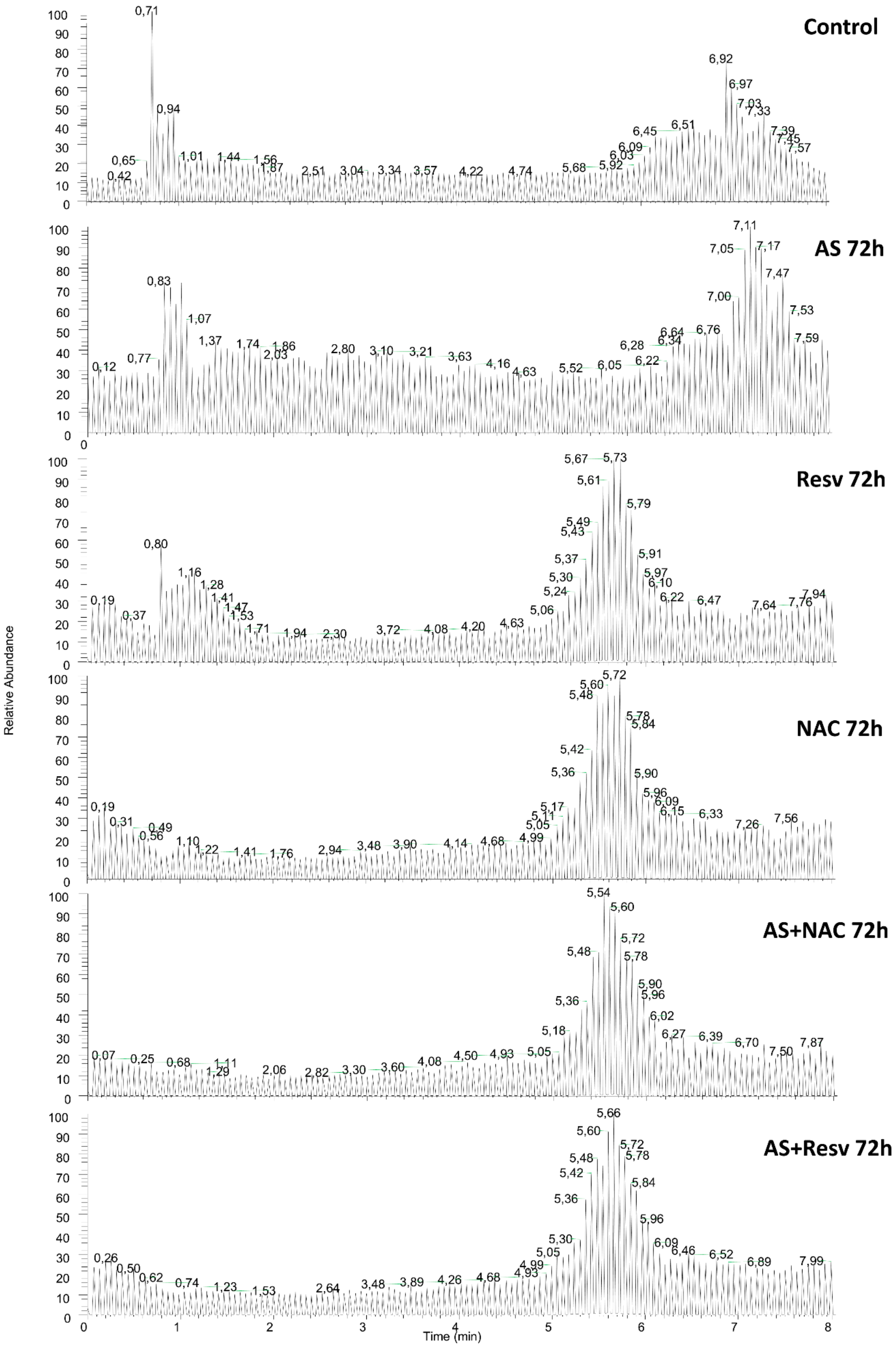
Control
0,71 0,94 0,65 0,42 1,01 1,44 1,56 1,87 2,51 3,04 3,34 3,57 4,22 4,74 5,68 5,92 6,03 6,09 6,45 6,51 6,92 6,97 7,03 7,33 7,39 7,45 7,57
AS 72h
0,12 0,77 0,83 1,07 1,37 1,74 1,86 2,03 2,80 3,10 3,21 3,63 4,16 4,63 5,52 6,05 6,22 6,28 6,34 6,64 6,76 7,00 7,05 7,11 7,17 7,47 7,53 7,59
Resv 72h
0,19 0,37 0,80 1,16 1,28 1,41 1,47 1,53 1,71 1,94 2,30 3,72 4,08 4,20 4,63 5,06 5,24 5,30 5,37 5,43 5,49 5,61 5,67 5,73 5,79 5,91 5,97 6,10 6,22 6,47 7,64 7,76 7,94
NAC 72h
0,19 0,31 0,49 0,56 1,10 1,22 1,41 1,76 2,94 3,48 3,90 4,14 4,68 4,99 5,05 5,11 5,17 5,36 5,42 5,48 5,60 5,72 5,78 5,84 5,90 5,96 6,09 6,15 6,33 7,26 7,56
AS+NAC 72h
0,07 0,25 0,68 1,11 1,29 2,06 2,82 3,30 3,60 4,08 4,50 4,93 5,05 5,18 5,36 5,48 5,54 5,60 5,72 5,78 5,90 5,96 6,02 6,27 6,39 6,70 7,50 7,87
AS+Resv 72h
0,26 0,50 0,62 0,74 1,23 1,53 2,64 3,48 3,89 4,26 4,68 4,93 4,99 5,05 5,30 5,36 5,42 5,48 5,60 5,66 5,72 5,78 5,84 5,96 6,09 6,46 6,52 6,89 7,99
Relative Abundance
Time (min)

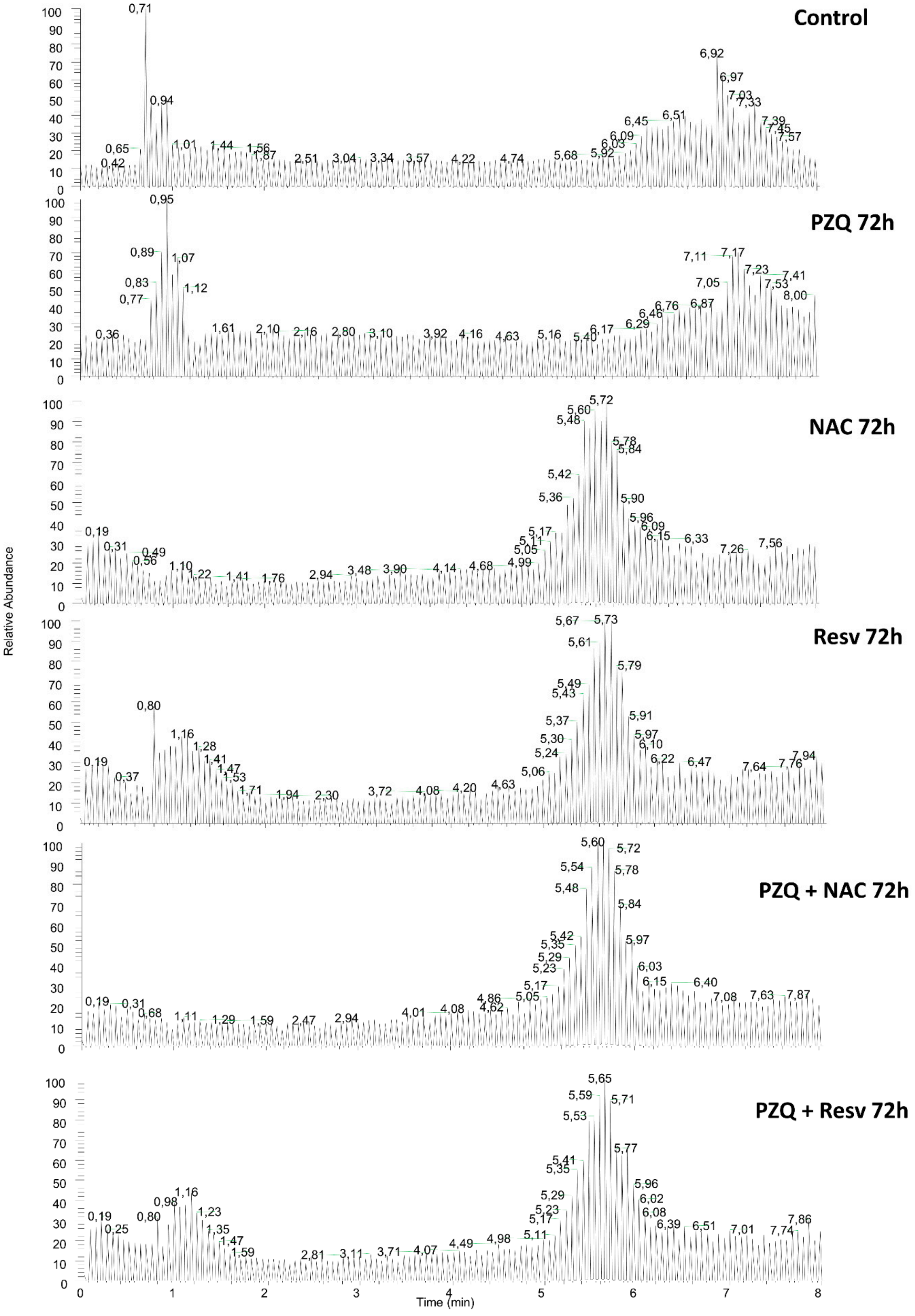
Control
0,71
0,94
0,65
0,42
1,01
1,44
1,56
1,87
2,51
3,04
3,34
3,57
4,22
4,74
5,68
5,92
6,03
6,09
6,45
6,51
6,92
6,97
7,03
7,33
7,39
7,45
7,57
PZQ 72h
0,95
0,89
1,07
0,83
1,12
0,77
0,36
1,61
2,10
2,16
2,80
3,10
3,92
4,16
4,63
5,16
5,40
6,17
6,29
6,46
6,76
6,87
7,05
7,11
7,17
7,23
7,41
7,53
8,00
NAC 72h
5,72
5,60
5,48
5,78
5,84
5,42
5,36
5,90
5,96
6,09
6,15
6,33
5,17
5,11
5,05
4,99
0,19
0,31
0,49
0,56
1,10
1,22
1,41
1,76
2,94
3,48
3,90
4,14
4,68
7,26
7,56
Resv 72h
5,67
5,73
5,61
5,79
5,49
5,43
5,37
5,30
5,24
5,06
5,91
5,97
6,10
6,22
6,47
7,64
7,76
7,94
0,80
1,16
1,28
1,41
1,47
1,53
1,71
1,94
2,30
0,19
0,37
3,72
4,08
4,20
4,63
PZQ + NAC 72h
5,60
5,72
5,54
5,78
5,48
5,84
5,42
5,35
5,29
5,23
5,17
5,05
4,86
4,62
5,97
6,03
6,15
6,40
7,08
7,63
7,87
0,19
0,31
0,68
1,11
1,29
1,59
2,47
2,94
4,01
4,08
PZQ + Resv 72h
5,65
5,59
5,71
5,53
5,77
5,41
5,35
5,29
5,23
5,17
5,11
5,96
6,02
6,08
6,39
6,51
7,01
7,74
7,86
0,19
0,25
0,80
0,98
1,16
1,23
1,35
1,47
1,59
2,81
3,11
3,71
4,07
4,49
4,98
Relative Abundance
Time (min)

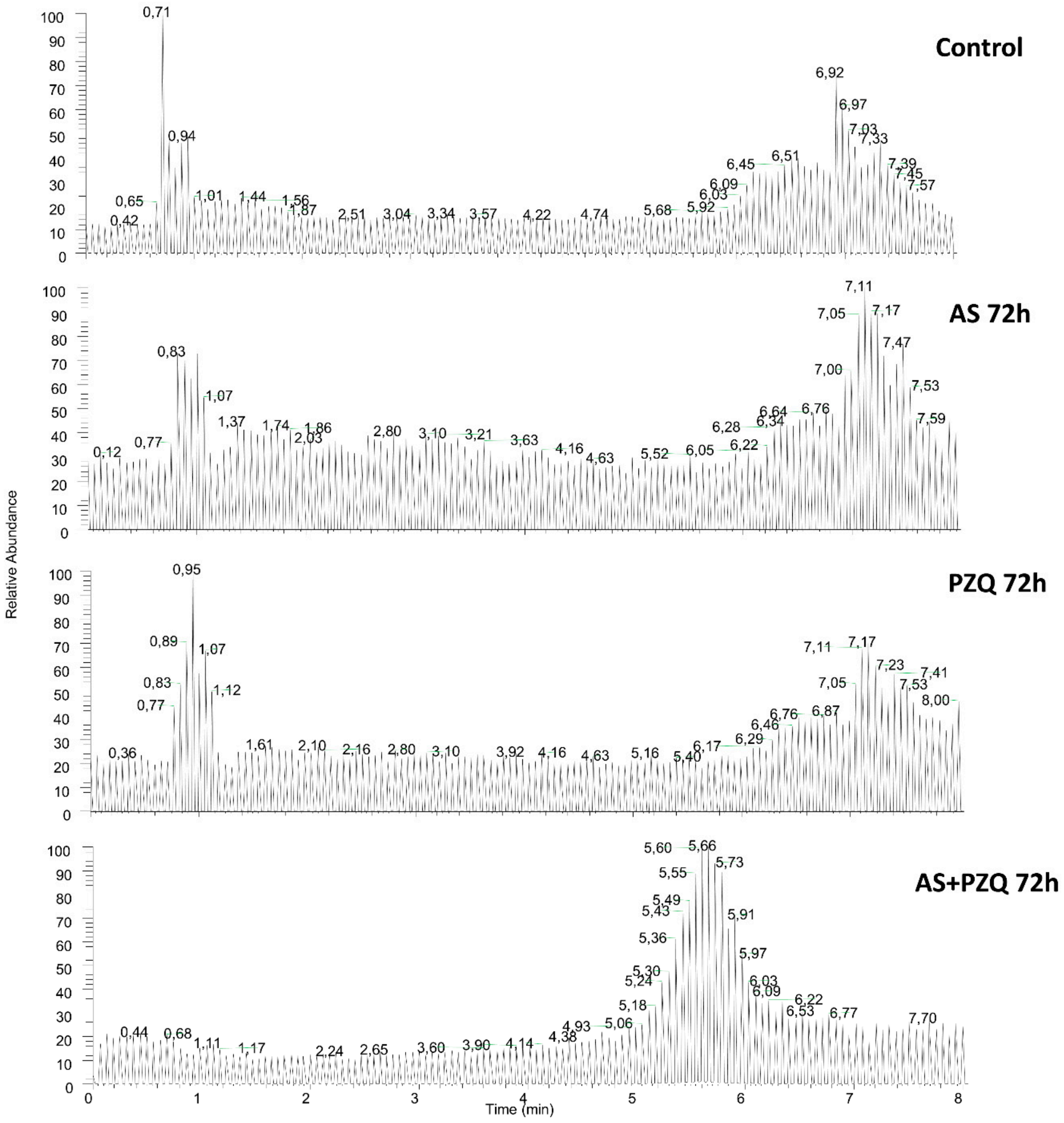
Control
AS 72h
PZQ 72h
AS+PZQ 72h
Relative Abundance
Time (min)

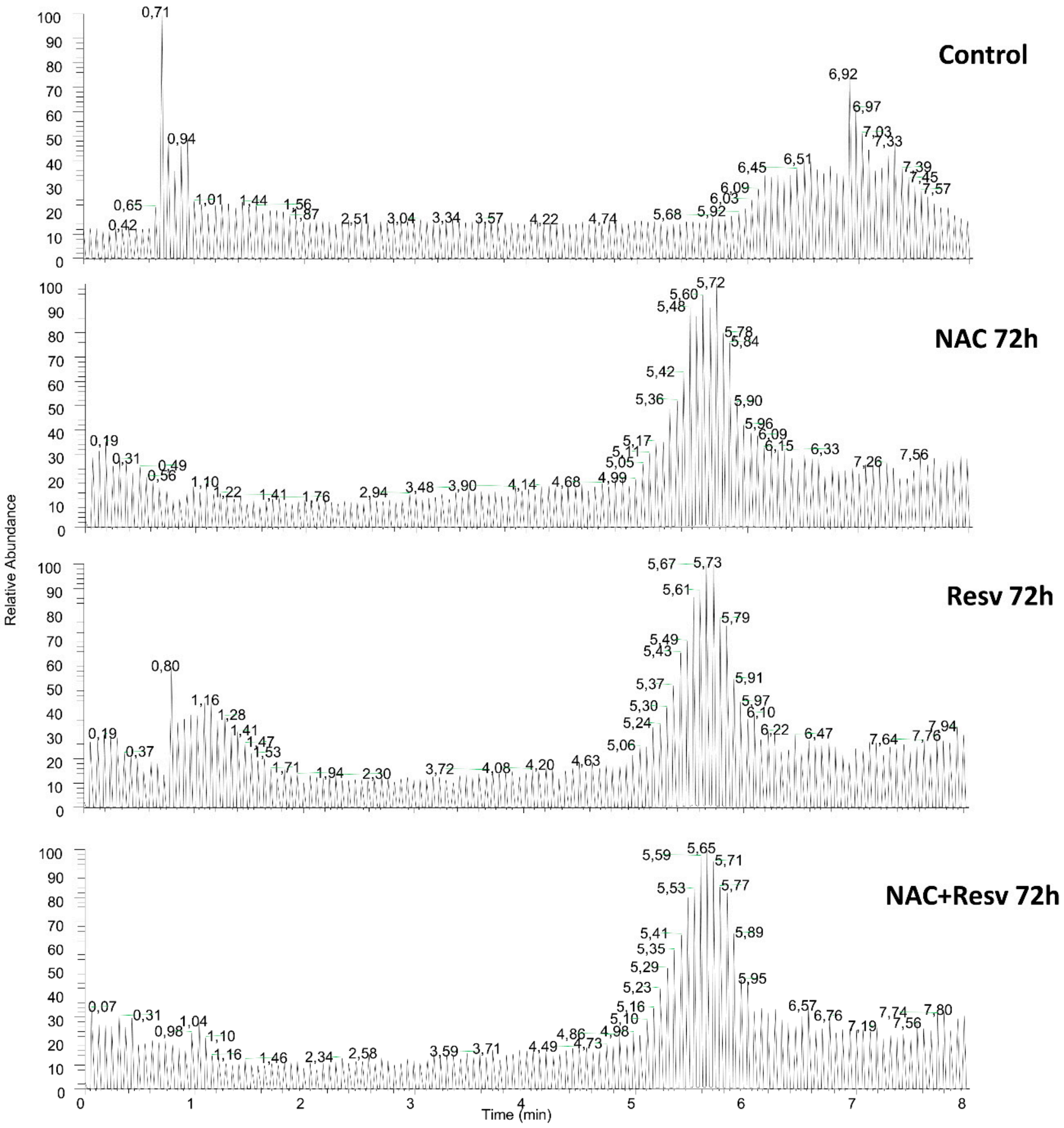
Control
NAC 72h
Resv 72h
NAC+Resv 72h
Relative Abundance
Time (min)
0,71
0,94
0,65
0,42
1,01
1,44
1,56
1,87
2,51
3,04
3,34
3,57
4,22
4,74
5,68
5,92
6,03
6,09
6,45
6,51
6,92
6,97
7,03
7,33
7,39
7,45
7,57
5,72
5,60
5,48
5,78
5,84
5,42
5,36
5,90
5,96
6,09
6,15
6,33
7,26
7,56
0,19
0,31
0,49
0,56
1,10
1,22
1,41
1,76
2,94
3,48
3,90
4,14
4,68
4,99
5,05
5,11
5,17
5,67
5,73
5,61
5,79
5,49
5,43
5,37
5,30
5,24
5,06
0,80
1,16
1,28
1,41
1,47
1,53
1,71
1,94
2,30
3,72
4,08
4,20
4,63
0,37
5,91
5,97
6,10
6,22
6,47
7,64
7,76
7,94
5,59
5,65
5,71
5,53
5,77
5,41
5,35
5,89
5,29
5,23
5,95
5,16
5,10
4,98
4,86
4,73
4,49
3,59
3,71
2,34
2,58
1,46
1,16
1,10
1,04
0,98
0,31
0,07
6,57
6,76
7,19
7,74
7,56
7,80

Sample	m/z
**Control** (starting compounds plus enzymes)	184.00; 186.96; 188.02; 194.99; 195.02; 199.17; 204.00; 208.13; 208.98; 214.09; 217.18; 220.99; 225.01; 227.13; 240.99; 247.17; 257.98; 271.19; 279.16; 391.28; 319.00; 190.91; 226.95; 242.93; 258.90; 288.92; 294.94; 310.91; 326.89; **362.93**; **378.90**; 394.87; 430.91; 446.89; 462.86; 498.90; 514.88; 582.86; 650.85; 202.10; 218.94; 240.92; 256.90; 272.87; 332.89; **338.90**; 354.88; 370.85; **414.9**0; **436.88**; **452.85**; 468.83; **474**.83; **512.79;** 534.85; **550**.83; **572**.81; **632.83**; **648.81**; 198.98; 220.26; 226.91; 256.90; 277.87; 278.88; 292.96; 294.85; 318.94; **346.80**; 354.88; 370.85; **376.86**; 390.93; 392.83; 410.90; 414.90; 452.85; 468.83; 474.83; **496.81;** **594.78**; **610.77**; **746.78**; 198.98; 256.90; 278.88; 318.94; 332.89; 354.87; 376.86; 390.93; **416.91**; 430.87; 436.88; 452.85; 474.83; 488.91; **490.79;** 508.87; **588.79**; 606.85; **670.79**; 202.18; 276.98; 292.96; 312.92; 370.97; 374.96; 198.98; 256.90; 292.96; 390.94; **528.84**; **626.81**; **730.81;** **768.70;** 214.94; 226.01; 466.32; **488**.30; 288.25; 391.28; **482.20**; 500.30; **522.29**
**AS 72h**	188.02; 194.99; 199.17; 204.00; 208.98; 209.01; 225.01; 240.99; 249.21; 257.98; 273.00; 279.16; 319.00; 391.28; 267.16; 190.91; 226.95; 242.93; 258.90; 294.94; 310.91; 326.89; **362.93**; **378.90**; 394.87; 430.91; 446.89; 462.86; 498.90; 514.88; 530.85; 582.86; 650.85; 180.90; 196.88; 232.92; 240.92; 242.92; 248.89; 256.90; 262.91; 264.86; 278.88; 284.89; 294.85; 300.86; 316.84; 316.88; 332.85; **338.90**; 368,85; 382.87; 398.84; 198.98; 220.26; 262,91; 272.87; 292.96; 318.94; 354.87; 370.85; **376.86**; 390.93; 410.91; **414.89**; **436.88**; **452.85**; **474.8**3; **490.79;** 495.81; **496.81;** **550.83;** **594.78;** **610.77;** **632.83;** 394.93; **416.91**; 488.91; 492.91; 508.87; **512.79**; **588.79**; 606.85; **730.81**; **768.70**; 468.82; 276.98; 468.82; 374.96; 394.92; 312.92; **626.81**; 218.94; **528.84**; 390.94; 303.02; 257.98; 288.25; 307.15; 343.30; 345.17; 212.58; 268.16; 291.64; 466.32; 467.32; **488.30**; 256.30; 500.30; 304.30; **482**.29; **522.29**;
**PZQ 72h**	188.02; 194.99; 204.00; 208.98; 225.01; 240.99; 249.21; 257.98; 271.19; 273.00; 303.02; 313.19; 319.00; 180.90; 210.93; 223.06; 226.06; 232.92; 240.92; 242.92; 248.89; 262.91; 278.88; 284.89; 300.90; 310.91; 316.88; 352.88; 368.89; **436.88**; **452.85**; 196.88; 264.86; 300.86; 368.85; 382.86; 398.84; 420.82; 181.01; 220.96; 236.93; 256.90; 318.94; 354.87; 360.88; 370.97; **376.86**; **416.91**; 458.86; **474.8**3; 480.84; **496**.81; **512.79;** **572.79**; **588.79**; **594.78**; **610.77**; **632.83**; **670.79;** **746.78**; **768.70**; 226.91; 288.95; 390.93; 292.96; 198.98; 218.94; **338.9**0; 394.92; 410.90; 414.89; 508.87; 534.85; **550**.83; 276.98; 312.92; 374.96; 488.91; **490.79;** 606.85; **626.81**; 492.90; **528.84**; **648.81**; **730.81**; 390.94; 508.88; 466.32; 467.32; **488.3**0; 288.25; 500.30; **522.29**
**NAC 72h**	219.65; 220.15; 232.17; 240.16; 240.66; 279.16; 304.30; 340.26; 340.76; 359.23; 378.29; 378.79; 396.80; 443.27; 475.33; 588.41; 679.51; 701.49; 702.50; **338.90**; **378.79**; 397.30; 453.34; **490.79;** 283.72; 273.63; 415.77; 199.17; 214.09; 566.43; 184.00; 217.18; 213.02; 218.97; 273.17; 186.96; 188.02; 194.99; 204.00; 218.98; 223.96; 240.99; **346.87**; **572.79**; **588.79**; **648.81**; 981.41; 207.98; 260.95; 275.93; 306.26; 203.00; 225.20; 1327.91; 188.06; 190.14; 227.13; 981.40; 200.97; 391.28; 410.90; 198.34; 195.09; 199.03; 255.94; 793.35; 182.98; 239.07; 397.80; 453.84; 200.8; 200.24; 280.13; 301.14; 230.25;
**Resv 72h**	219.65; 220.15; 232.17; 240.16; 279.16; 304.30; 340.26; 340.76; 359.23; 378.29; 378.79; 396.80; 443.27; 475.33; 588.41; 589.41; 679.51; 701.49; 702.50; 199.17; 397.30; 415.30; 240.66; 283.72; 453.34; 566.43; 415.77; 184.00; 214.09; 217.18; 453.34; 453.84; 218.97; 273.63; 203.22; 220.93, 226.95; 814.58; 190.91; 242.93; 258.90; 294.94; 310.91; 326.89; **362.93;** **378.90**; 394.87; 430.91; 446.89; 462.86; **512.79;** 514.87; 530.85; **550.83**; **572.79**; 582.86; **594.78;** 598.84; **626.81**; 650.85; 717.47; **746.78**; **768.70**; 242.92; 240.92; 256.90; 262.91; 278.88; 292.96; 294.85; 300.86; 312.92; **338.9**0; 354.88; 360.88; **376.86**; 382.87; 398.84; 410.90; **436.88**; 458.86; **474.8**3; **496**.82; **588.79;** **610.77**; **670.79;** **730.81**; 390.94; 394.92; **414.9**0; 430.86; **452.85**; 474.84; 508.88; 534.86; 196.96; 275.93; 332.89; **528.84**; 606.85; 626.82; **632.83**; 214.94; 218.98; 255.97; 332.88; 374.96; 223.96; 259.96; 260.95; 373.91; 234.91; 207.99; 204.00; 186.96; 275.92; 227.13; 264.99; 306.26; 391.28; 188.02; 188.06; 203.00; 225.20; 182.98; 200.97; 207.98; 213.02; 1327.96; 198.34; 188.06; 793.36; 680.52; 397.80; 453.85; 301.14; 230.25

m/z 466.32 correspond to glycocholic acid; m/z 313.19 correspond to PZQ.

Sample	m/z
**AS+NAC 72h**	199.17; 219.65; 279.16; 283.72; 304.30; 340.26; 340.76; 354.23; **378.90**; 396.80; 397.30; 453.34; 566.43; 679.51; 680.51; 701.49; 184.00; 214.09; 217.18; 415.77; 279.16; 190.14; 199.02; 213.02; 218.97; 255.94; 306.26; 188.02; 194.99; 204.00; 225.01; 225.20; 240.99; 184.00; 200.97; 273.63; 391.28; 186.01; 188.06; 203.00; 227.13; 527.78; **588.79; 670.79; 746.78; 768.70;** 182.98; 239.07; 397.80; 453.84; **512.79; 528.84;** 200.08; 301.14; 588.41; 280.16; 230.25;
**AS+Resv 72h**	1991.7; 219.65; 220.15; 232.17; 240.16; 279.16; 304.30; 340.26; 340.76; 359.23; 378.29; **378.79**; 396.80; 397.30; 443.27; 475.33; **588**.41; **670.79**; 679.51; 701.49; 702.50; 283.72; 453.34; 566.43; 214.09; 184.00; 217.18; 415.77; 213.09; 218.97; 306.26; 391.28; 1372.72; 186.96; 190.14; 204.00; 223.96; 255.94; 273.63; 203.00; 981.40; 188.06; 227.13; 186.01; 200.97; 225.20; 239.07; 215.16; 793.32; 1327.78; 397.80; 453.84; 200.08; 280.16; 301.14; 230.25: 284.33;
**PZQ+NAC 72h**	199.17; 219.65; 232.17; 240.16; 273.63; 279.16; 283.72; 340.26; 340.76; 359.23; 396.80; 397.30; 415.77; 443.27; 453.34; 566.43; **588**.41; 679.51; 701.49; 184.00; 214.09; 217.18; 218.97; **338.90**; 306.26; 453.84; **482.20**; **496.81**; **572.79; 594.78; 610.77; 632.83; 730.81; 746.78;** 186.96; 190.14; 213.02; 225.20; 255.94; 223.96; 793.31; 1327.79; 198.32; 203.00; 227.13; 793.30; 188.06; 204.00; 391.28; 200.97; 215.16; 981.40; 186.01; 215.16; 182.98; 239.07; 397.80; 680.52; 453.85; 488.30; 200.08; 200.24; 280.16; 301.14;
**PZQ+ Resv 72h**	219.65; 220.15; 232.17; 240.16; 279.16; 340.26; 340.76; 359.23; 378.29; **378.79**; 396.80; 415.30; 443.27; 475.32; 557.44; **588.41**; 679.51; 701.49; 702.50; 240.66; 283.72; 397.30; 453.34; 475.33; 199.17; 214.09; 217.18; 415.77; **474.83**; 566.43; 193.97; 226.95; 242.93; 604.38; 680.52; 717.47; 814.58; 190.91; 242.91; 258.90; 304.90; 310.91; 326.89; **362.93**; **378.90**; 394.87; **436.80**; 446.89; 462.86; **488.30**; **496.81**; **550.83**; **670.79**; 718.47; 204.00; 220.93; 327.19; 491.30; 214.94; 240.92; 240.99; 255.97; 256.90; 262.91; 276.98; 278.88; 292.96; 312.92; **338.90**; 354.88; 370.97; 374.96; 390.94; 394.92; 410.90; 508.88; **414.90**; 430.86; **512.79**; **528.84**; **572.79**; **594.78**; 606.85; **610.77; 626.81**; **746.78**; **452.83**; **482.20**; 488.91; **522.29**; 218.98; 260.95; 275.93; 332.88; 223.96; 207.99; 259.96; 234.91; 188.02; 186.96; 207.98; 218.97; 194.99; 275.92; 306.26; 273.63; 391.28; 203.00; 200.97; 227.13; 213.02; 184.00; 198.33; 225.20; 793.34; 182.98; 188.06; 255.94; 397.80; 453.84; 466.32; 200.24; 200.08; 301.14; 313.19; 214.25;
**AS+PZQ 72h**	199.17; 219.65; 240.16; 279.16; 283.72; 301.14; 304.30; 340.26; 340.76; **346.80;** 352.34; 359.23; 396.80; 397.30; 401.29; 415.30; **416.91**; 445.31; 459.33; **482.20; 550.83; 588**.41; 679.51; 701.49; 273.63; 371.28; 443.27; 453.34; 214.09; 359.73; 415.77; 475.33; **488.30; 496.81**; 566.43; **594.78**; **648.81; 670.79**; **730.81; 746.78**; 214.09; 415.77; 566.43; 680.51; 217.18; 981.41; 184.00; 397.80; 186.96; 190.14; 213.04; 218.97; 306.26; 223.96; 203.00; 225.20; 255.94; 793.27; 1327.88; 188.06; 227.13; 198.32; 391.28; **768.70;** 793.29; 200.97; 195.09; 199.03; 527.78; 182.98; 215.16; 186.01; 453.84; 415.35; (313.14, pzq); 200.08; 200.24; 280.16; 230.25;
**NAC+ Resv 72h**	219.65; 220.15; 232.17; 240.16; 279.16; 301.14; 340.26; 340.76; 359.23; **378.29**; 396.80; 401.29; 415.30; **436.80**; 443.27, **452.85**; **482.20; 496.81; 528.84; 588**.41, **626.81**; **632.83**; 679.51; 701.49; 702.50; 475.33; 589.41; 240.66; **416.91**; 453.34; 370.30; **376.86**; 397.30; 199.17; 283.72; 359.73; 214.09; 217.18; 415.77; 566.43; 184.00; 680.51; 188.02; 204.00; 214.94; 218.98; 223.96; 240.99; 255.97; 260.95; 275.93; 292.96; 312.92; 390.94; 410.90; 207.99; 218.97; 225.20; 306.26; 186.96; 203.0; 213.02; 227.13; 273.63; 391.28; 186.01; 198.34; 255.94; 793.36; 188.06; 200.97; 239.07; 182.98; 527.18; 397.80; 453.84; 475.32; 200.08;
